# Nonossifying fibroma of the lower femur with genu valgum: a case report

**DOI:** 10.1186/s12887-023-04224-6

**Published:** 2023-10-23

**Authors:** Zhibing Dai, Maierdanjiang Maihemuti, Yachao Sun, Renbing Jiang

**Affiliations:** Affiliated Tumor Hospital of Xinjiang Medical University, Urumqi, China

**Keywords:** Femur, Metaphysis segment, Nonossifying fibroma, Genu valgum, Child

## Abstract

**Background:**

Nonossifying fibroma is common in children and adolescents, and nonossifying fibroma with genu valgum is rare in the clinic. This article evaluated the effectiveness of treatment in a case of nonossifying fibroma of the lower femur with genu valgum.

**Case presentation:**

A 16-year-old girl complained of pain in the lower part of her right thigh for one year. She was diagnosed as non ossifying fibroma of the right femur with secondary valgus deformity of the right knee, and was treated in our hospital. We performed curettage, bone grafting and internal fixation,and corrected the valgum deformity at the same time. The patient's incision healed well, the pain was disappeared, and the mechanical axis of lower limbs was corrected. No tumor recurrence was found on X- ray examination one year after operation, and the fracture end was healed. The patient could walk normally, and she was satisfied with her limb function.

**Conclusion:**

Nonossifying fibroma with genu valgum is rare in the clinic. The patient was satisfied with our treatment, which achieved a good curative effect.

## Background

Nonossifying fibroma is common in children and adolescents, mostly in the distal femur, proximal tibia and distal tibia. It originates from one side of the cortex and is indicated by osteolytic destruction with clear edges and sclerotic edges. Nonossifying fibroma is a benign lesion. Most people have no symptoms, which are often found on plain radiographs for other reasons [1]. Generally, it does not affect daily life and does not need special treatment. However, we have found that some patients have local pain, the focus is visible on imaging examination, and there is a risk of pathological fracture,and then surgical treatment is needed in this case.

## Case presentation

**Fig. 1:**
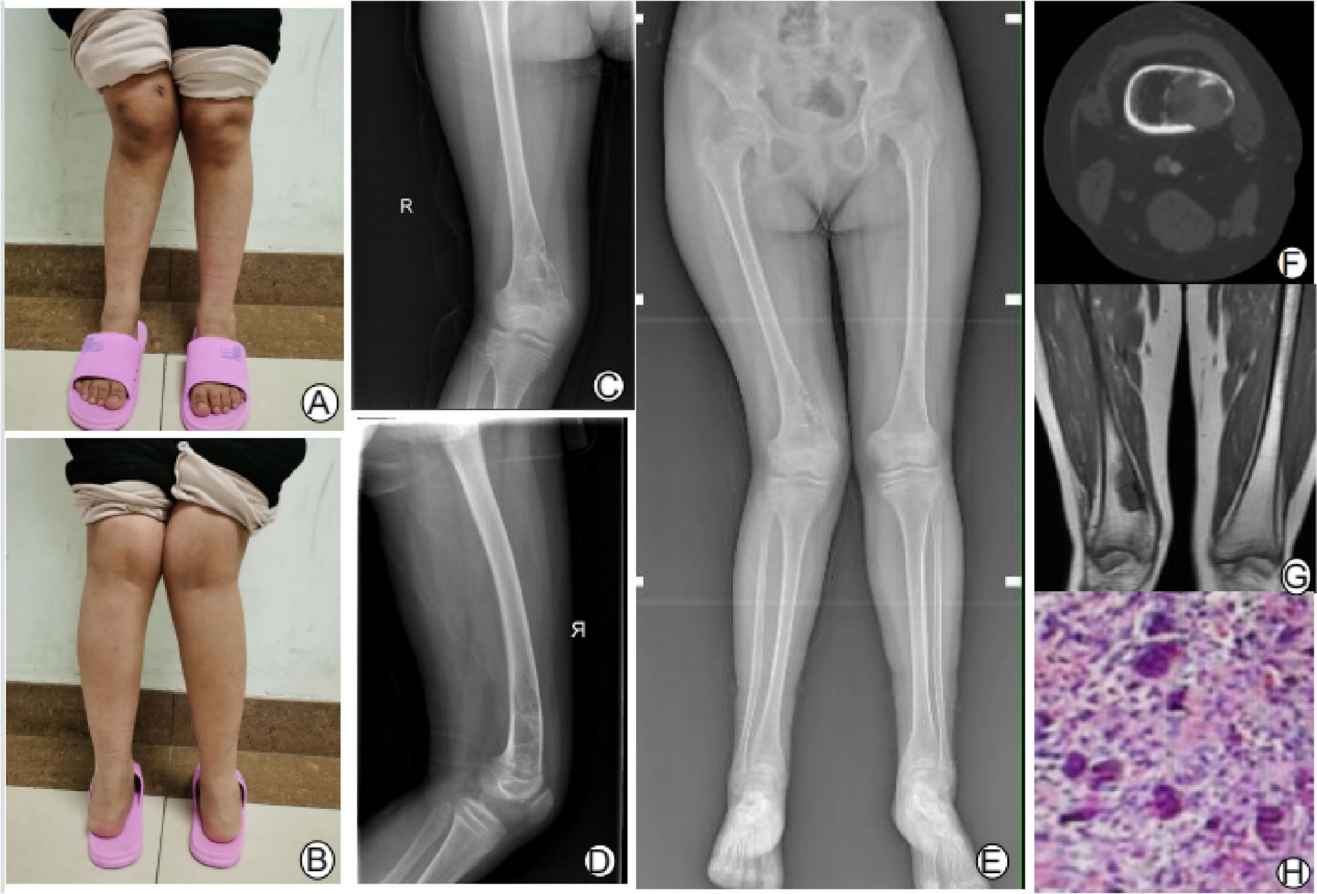
**Ⓐ**-**Ⓑ** Genu valgum deformity of the right lower limb. **Ⓒ**-**Ⓔ** X-ray showed partial osteolytic destruction of the metaphyseal segment of the femur, destruction of the medial cortex, and valgum deformity of the knee. X-ray of the whole length of the lower limb showed valgum deformity of the right knee. **Ⓕ** CT showed partial osteolytic destruction and cortical destruction of the femur, with clear boundaries and sclerotic edges. **Ⓖ** T1 imaging showed a low signal in the medullary cavity at the metaphyseal segment of femoral with clear boundaries. **Ⓗ** Spindle cell lesions and more multinucleated giant cells were observed in the preoperative puncture pathology images

A 16-year-old girl complained of pain in her right knee for one year and claudication while walking for two weeks. The patient said that pain had gradually developed in the lower part of the thigh one year ago. The pain increased during walking and was relieved after rest. Physical examination showed that the right knee joint had a valgum deformity (Fig. 1A, B), the lower part of the right thigh did not touch an obvious mass, the inner side of the lower part of the right thigh had local tenderness, and the flexion and extension function of the knee joint was acceptable. X-ray showed valgum deformity in the right knee, osteolytic bone destruction in the metaphyseal segment of the right femoral shaft, discontinuity of the medial cortex and sclerosis at the edge of the lesion, and separation could be seen inside. We classified the knee valgum deformity according to Ranawat [2]. On the full-length positive X-ray of both lower limbs, a femoral tibial angle < 10° is type I, a femoral tibial angle of 10°-20° is type II, and a femoral tibial angle > 20° is type III. The femoral tibial angle of this patient was 19.7°, which belonged to Ranawat type II (Fig. 1E). It can be seen that the eccentric, osteolytic and well-defined focus of the femoral metaphysis on the X-ray (Fig. 1C, D). CT showed that the medial bone cortex at the metaphyseal segment of the femur was fractured, with eccentric osteolytic destruction and sclerotic margins (Fig. 1F). MRI showed a low signal in the metaphyseal segment of the femur, a fractured medial cortex and a clear edge on the T1 image (Fig. 1G). Puncture pathology showed that nonossifying fibroma was possible but that giant cell tumor of bone could not be excluded (Fig. 1H). The patient was young and had a long history. The focus originated from one side of the bone cortex, and map-like eccentric osteolytic destruction was present at the metaphyseal segment of femur, with a clear boundary, sclerotic edge, no involvement at the bone end, separation in the focus, and secondary valgum deformity. It was more likely to be non ossifying fibroma after multidisciplinary discussion. The operation plan was curettage, osteotomy, orthopedic bone grafting and internal fixation.

**Fig. 2:**
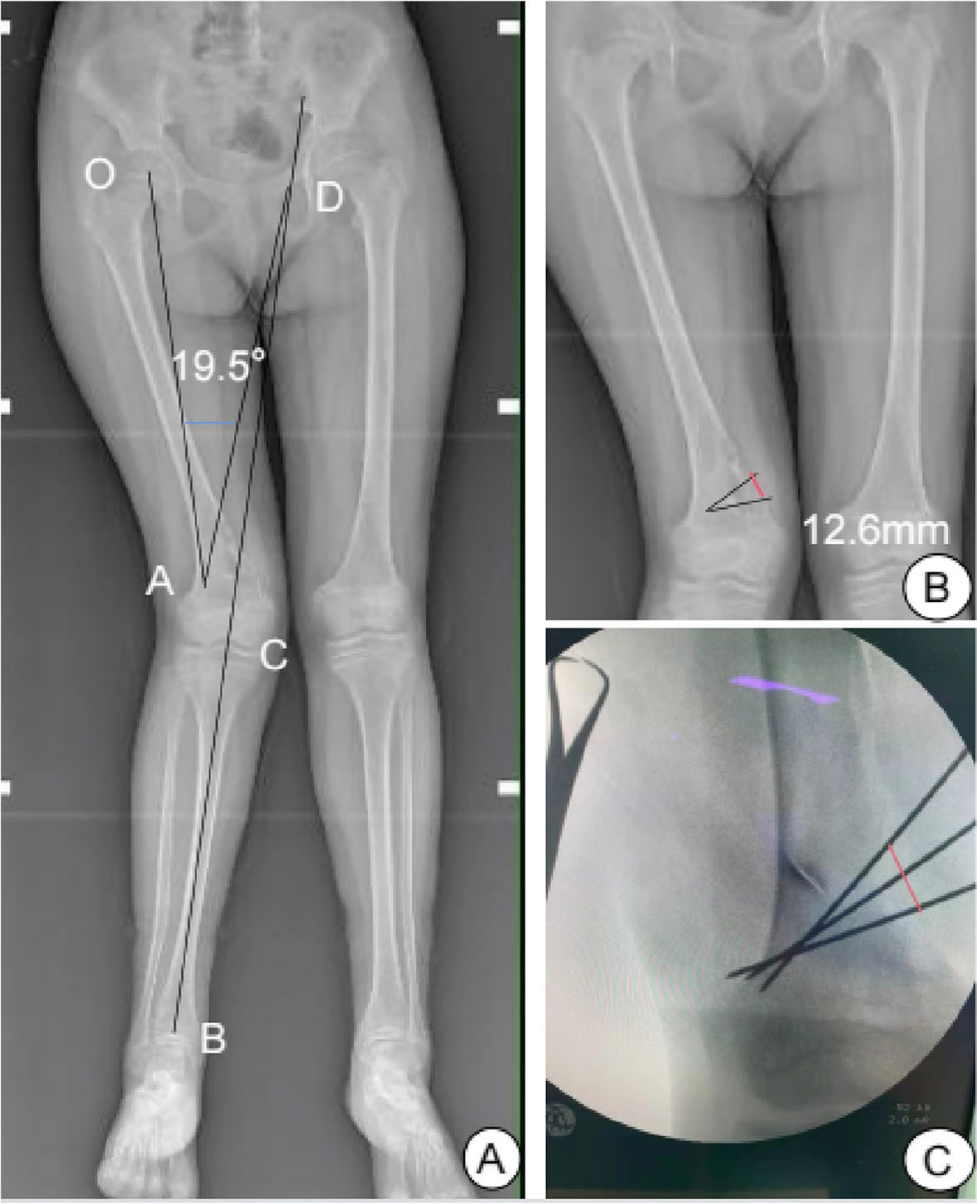
**Ⓐ** Planning the osteotomy angle and osteotomy direction. **Ⓑ** The length of the osteotomy was 12.6 mm. **Ⓒ** Confirming the osteotomy length during the operation (marked with red line)

We completed the relevant preoperative situation of the patient and planned to carry out expanded tumor curettage and osteotomy. The patient's valgum deformity was located in the distal femur, and there was no deformity of the tibia. The amount and direction of osteotomy were designed according to the Miniaci [3] method. The ankle joint center (B) and knee joint center (C) were connected on the full-length film of both lower limbs, and the line was extended to the proximal end. The position of the osteotomy hinge point (A) was 5 mm above the lateral condyle of the femur and approximately 5–10 mm inward from the lateral cortex. This point was the center of the circle (A). The line was rotated between this point (A) and the center of the femoral head (O) and intersected the extension of line BC at D. The rotation angle (∠ OAD = 19.5°) was the osteotomy degree (Fig. 2A). The hinge point was taken as the apex, and an isosceles triangle was made toward the medial cortex of the distal femur. The top angle of the triangle was the osteotomy degree mentioned above, and the length of the bottom edge was 1.26 cm, which was the length of the medial cortex of the femur to be corrected (Fig. 2B).

**Fig. 3:**
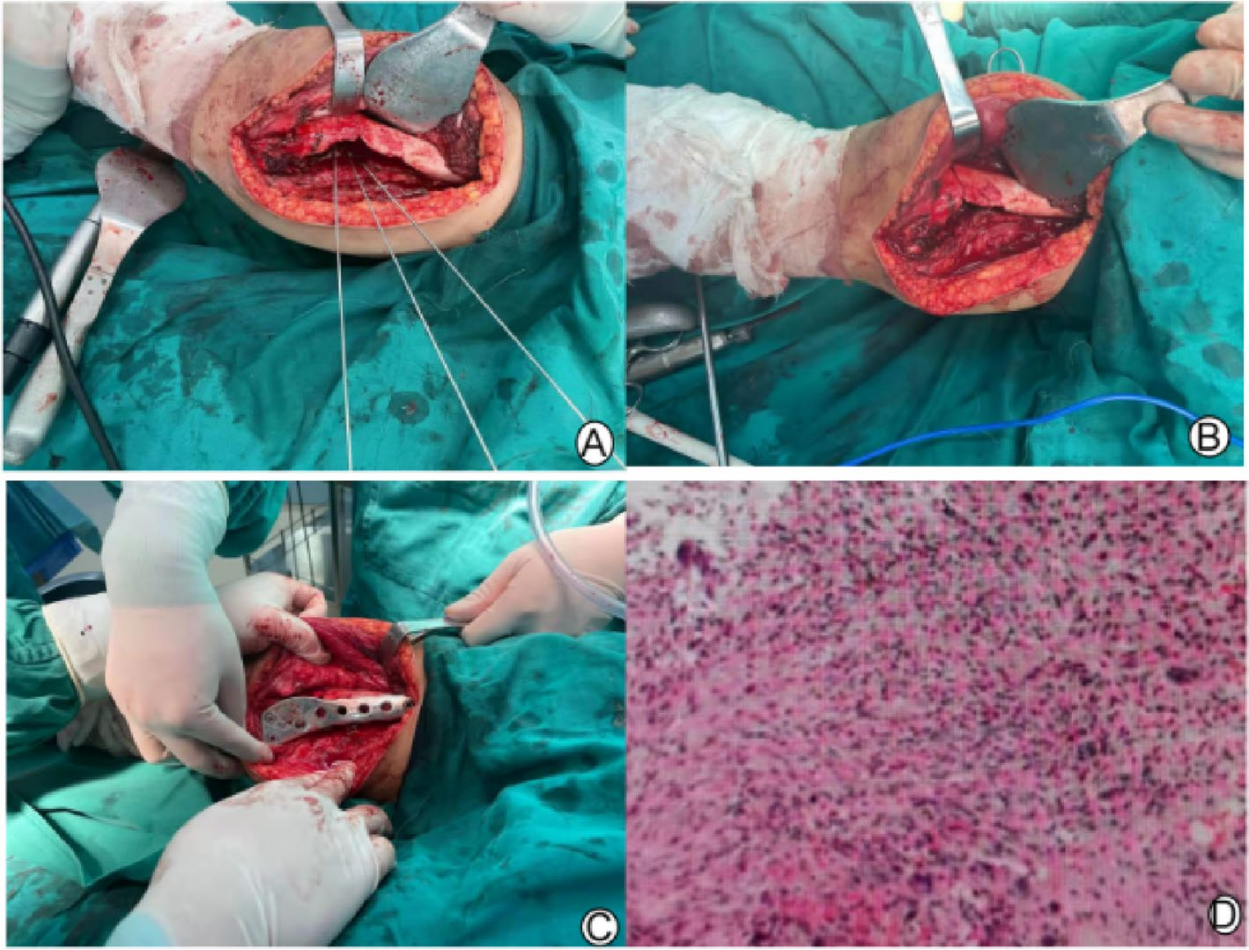
**Ⓐ** After curettage, Kirschner wire was used to locate the range of the osteotomy. **Ⓑ** Fracture closure after osteotomy. **Ⓒ** Medial plate fixation and allogeneic bone grafting. **Ⓓ** Postoperative pathology showed giant cell lesions, proliferation of stromal spindle cells, local bleeding and new bone formation

We made a medial incision in the distal femur. An oval window was opened on the medial bone cortex, with a window area of approximately 5 × 1 cm. The diseased tissue in the medullary cavity was scraped off, the medullary cavity was ground with a grinding drill, the medullary cavity was burned with an electric knife, and finally, the tissue was soaked in anhydrous alcohol for five minutes. According to the preoperative design, two Kirschner wires were inserted and served as the osteotomy positioning guide needle. The angle was checked via fluoroscopy and determined to be too small; then, another Kirschner wire was implanted. On fluoroscopy, the osteotomy angle was good. The outermost two Kirschner wires were equivalent to two sides of the isosceles triangle planned in the preoperative design. The Kirschner wires intersected 5–10 mm inside the cortex of the lateral condyle of the femur, which was the hinge point of the osteotomy (Fig. 3A). The distance between the two Kirschner wires (red lines on Fig. 2C) was the bottom edge of the isosceles triangle planned preoperatively and was approximately 1.26 cm (Fig. 2C). A wedge-shaped osteotomy was made with a pendulum saw was used to control the osteotomy depth, stopping when it was close to the contralateral cortex and protecting the contralateral hinge; then the osteotomy end was closed slowly (Fig. 3B). After fluoroscopic examination indicated that the mechanical axis of right lower limb was satisfactory, the medial plate of the distal femur was implanted (Fig. 3C). The flexion and extension function of the knee joint was good, and the fracture end was stable. After washing, the broken end medullary cavity was implanted with allogeneic bone, and the drainage tube was placed and sutured layer by layer.

**Fig. 4:**
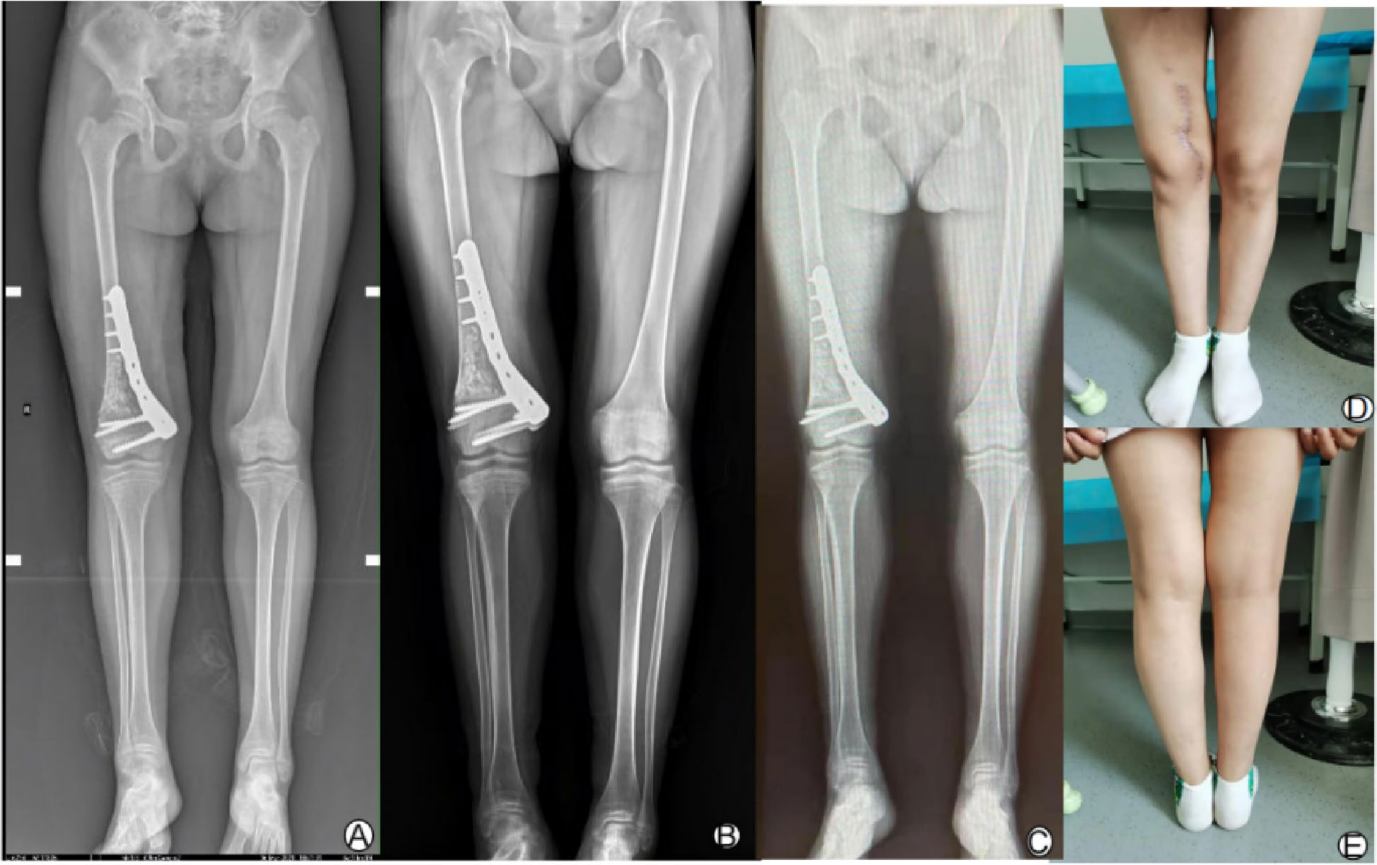
**Ⓐ** After the operation, the full-length X-ray showed that the mechanical axis of lower limb was corrected. **Ⓑ** Five months after the operation, X-ray showed that the lateral fracture line was blurred, the allogeneic bone particles in the medullary cavity were partially absorbed, and the mechanical axis of the lower limb was normal. No tumor recurrence was present. **Ⓒ** One year after the operation, X-ray showed that the lateral fracture line was completely healed, obvious osteogenesis was present in the medullary cavity, the mechanical axisof the lower limb was normal, and there was no tumor recurrence. **Ⓓ**-**Ⓔ** One year after the operation, and the mechanical axis of the lower limbs was normal by physical examination

The patient performed muscle strengthening by isometrically contracting the quadriceps femoris in the hospital bed. When there was no obvious pain, knee flexion and extension exercises were started. The incision was changed regularly to observe whether there was an infection. Postoperative pathology showed giant cell lesions, proliferation of stromal spindle cells, local bleeding and new bone formation (Fig. 3D). Combined with the medical history, the final diagnosis is considered nonossifying fibroma. The full-length X-ray of the lower limbs showed that the mechanical axis of right lower limb had been corrected. The femoral tibial angle was 5°, the range of motion of the knee was 0° -130°. There was no tumor recurrence five months after operation, X-ray showed that the lateral fracture line was blurred but not completely healed, and the bone graft in the medullary cavity was partially absorbed. One year after the operation, there was also no tumor recurrence, the lateral fracture line was completely healed, and obvious osteogenesis was present in the medullary cavity (Fig. 4).

## Discussion and conclusions

Nonossifying fibroma (NOF) is a common bone lesion in the clinic. Its histological features include a mixture of benign fibroblast proliferation and osteoclastic giant cells [4]. It is a nonneoplastic process and considered a developmental abnormalities [5]. The actual incidence rate of NOF is not clear. It has been estimated that approximately 30% of children have one or more lesions [6, 7]. Radiologically, this was an isolated, eccentric and osteolytic lesion of the metaphysis segment of the long shaft, which usually has a map-like shape. Usually, NOF is accidentally discovered on X-ray for other reasons. Most patients are asymptomatic, and a few NOF cases involve pain and swelling, especially with physical activity [8]. The disease is usually asymptomatic and has a good prognosis. Generally, it subsides naturally over time. Nevertheless, there is a risk of pathological fracture in some parts, depending on the size of the lesion. According to the classification system proposed by Ritschl [9], the natural course of nonossifying fibroma includes four stages. Stage A involves a small lesion with eccentricity near the bone cortex. Stage B involves large osteolytic lesions with thin sclerotic edges. Phase C lesions show an increased degree of sclerosis. Stage D lesions show complete sclerosis. Patients with stage B lesions have an increased risk of fractures. However, no fractures are found in stages A, C and D. In Ritschl [8] studies, the average length of fracture lesions was 44 ± 9 mm, the transverse section of the lesions accounted for an average of 75 ± 19% of the coronal plane of the medullary cavity, and the sagittal plane of the medullary cavity accounted for an average of 87 ± 13%. The location of the distal tibia seems to be associated with the risk of pathological fractures. Larger lesions are more likely to lead to fractures. Therefore, patients in stage B should be advised to reduce or avoid strenuous exercise to prevent fractures. Moreover, imaging changes in these patients should be closely monitored until the lesion reaches phase C [1]. Arata [10] described that there was a risk of pathological fracture when the lesion reached more than 50% of the transverse diameter of the medullary cavity or when the lesion length was 33 mm. The standard treatment method is lesion curettage and cancellous bone grafting. Internal fixation is required for impending or broken fractures.

NOF is common in the clinic, but pathological fractures with valgum deformity are rare. Considering the patient's long history, the large focus and the long-term presence of microfracture, the valgum deformity gradually worsened. Pathologic analysis is often needed to make the final diagnosis in combination with the medical history and imaging. The focus removal and limb orthopedics finally achieved good surgical results at the same time. But we still need to further follow up the limb function,and to see if it will recur in the long term.

## Data Availability

If anyone needs data, please contact Zhibing Dai, e-mail: zbd1137582180@163.com.

## Abbreviations

NOF: Nonossifying fibroma
CT: Computerized tomography
MRI: Magnetic resonance imaging
